# Mucocutaneous Leishmaniasis With Oral Involvement: A Case Report

**DOI:** 10.1155/crid/1229183

**Published:** 2025-08-22

**Authors:** Pierpaolo De Francesco, Paolo Vescovi, Claudio Feliciani, Roberta Iaria, Luigi Corcione, Ilaria Giovannacci

**Affiliations:** Department of Medicine and Surgery, University of Parma, Parma, Italy

**Keywords:** mucocutaneous leishmaniasis, oral leishmaniasis, oral pathology

## Abstract

The primary objective of the present study is the retrospective analysis of a clinical case of oral leishmaniasis treated at Parma Hospital and a review of the literature on mucosal leishmaniasis (ML) with oral cavity involvement. We report the case of a patient diagnosed in 2017 with mucocutaneous leishmaniasis who was referred to our clinic due to the emergence of oral manifestations. Through a detailed review of the clinical documentation, we aim to describe the clinical presentation, diagnosis, treatment choices made, and evolution of the disease. Literature describes various pharmacological approaches for leishmaniasis, with treatment selection dependent on clinical presentation. ML does not spontaneously resolve, and systemic therapy is necessary. Due to its rarity, oral leishmaniasis can be challenging to diagnose. This case report can help physicians recognize the clinical presentation of oral lesions, enabling prompt diagnosis and effective treatment.

## 1. Introduction

*Leishmania* is transmitted by approximately 70 different species of infected female phlebotomine sand flies [1, 2]; it is caused by various protozoan species within the genus *Leishmania*. It is the third most prevalent vector-borne disease worldwide, following malaria and African trypanosomiasis, and contributes significantly to the global disease burden. More specifically, it affects vast populations in tropical and subtropical areas all over the world [3]. Poverty, population migration, malnutrition, inadequate hygiene practices, and immunosuppression constitute significant risk factors for leishmaniasis [4]. The global incidence of leishmaniasis decreased substantially in the past decade: from between 200,000 and 400,000 new cases in 2012 to between 50,000 and 90,000 in 2017 [5]. The World Health Organization (WHO) estimates that leishmaniasis is endemic in almost 100 countries, and the estimated total risk population is approximately 350 million people [6]. Annually, 900,000 to 1.3 million new cases are reported, 20,000 to 30,000 deaths occur, and the overall prevalence is 12 million cases. In most countries, incidence figures are likely underreported due to a combination of unrecognized cases and nonmandatory reporting. This is particularly evident in many sub-Saharan African nations, where accurate burden estimation is challenging and, as a result, the implementation of crucial control measures is impeded [7]. Recently, an increased incidence of coinfection of *Leishmania* and human immunodeficiency virus (HIV) has been noted, partly attributed to the geographic overlap between the two diseases. This condition is considered a neglected infectious disease by the WHO [8]. The term *Leishmania*, which dates to 1903, comes from the Scottish pathologist William Boog Leishman, and there has been an increase in infection rates from various factors such as urbanization, climate change, increased migration, and travel to endemic areas for tourism purposes [9]. *Leishmania* species are typically classified into Old World and New World types based on their geographic distribution and the different vectors that act as reservoirs for the parasite. Old World leishmaniasis is prevalent in open, semiarid areas or deserts across parts of Asia, the Middle East, Africa (primarily in northern and tropical regions), and Southern Europe. In contrast, New World leishmaniasis is commonly found in forested areas of Mexico, Central, and South America. Recently, isolated cases of leishmaniasis have been reported in Southern Europe [10].

Leishmaniasis presents in three different clinical forms: visceral leishmaniasis (VL), cutaneous leishmaniasis (CL), and mucocutaneous leishmaniasis (MCL) [11–13]. American tegumentary leishmaniasis (ATL) can be classified as a fourth syndrome induced by New World *Leishmania* species characterized by CL and MCL manifestations; rarer forms, such as diffuse and disseminated CL, have also been documented [14]. However, a more comprehensive classification recognizes 11 distinct clinical forms.

MCL and pure ML forms can develop months or even years after exposure in endemic regions, often leading to delayed diagnosis due to this extended latency period.

All forms of leishmaniasis can manifest with initial mucosal lesions in the head and neck area, potentially affecting the oral cavity. Dentists may encounter patients with symptoms like difficulty swallowing, voice changes, and shortness of breath, which could be early signs of oral leishmaniasis. Leishmaniasis can affect the head and neck region with primary mucosal lesions that can imitate a wide range of oral infectious diseases and neoplasms [15].

Early diagnosis by dental professionals is crucial to prevent the disease from spreading systemically [16].

We lack evidence for potentially beneficial treatments. This can be partially attributed to the pharmaceutical industry's limited interest in developing novel antileishmanial therapies, as the disease is mainly endemic in remote areas and this often makes patient follow-up difficult, leading to high rates of study dropout and severely impacting data integrity [6]. Leishmaniasis treatment includes a range of modalities, such as topical and systemic medications, physical interventions, and immunotherapeutic strategies [17]. A 2019 Cochrane review found that antimonial drugs, particularly meglumine antimoniate, and oral miltefosine showed the strongest evidence for improving the clinical condition of leishmaniasis patients [18].

For these reasons, prevention of this disease whose diagnosis and therapeutic treatment are still unclear should play a crucial role. Leishmaniasis prevention strategies include reducing the number of infected sand flies (vector control), controlling the animal reservoirs of *Leishmania* in zoonotic areas, and developing effective human vaccines [19]. A 2015 Cochrane review shows that using insecticides to reduce phlebotomine sand fly numbers appears to be effective at reducing CL, while the effectiveness of these measures in reducing VL incidence remains unknown due to a lack of evidence [20].

To date, no clinically effective vaccine against any form of human leishmaniasis has been developed [21–23]. A major obstacle to developing an effective leishmaniasis vaccine is the lack of an appropriate adjuvant or delivery system. Recent years have witnessed significant advancements, culminating in the progression of four candidate vaccines toward clinical trials [24]:

• LEISHF3+ GLA-SE: A recombinant fusion protein administered with potent Th1-inducing adjuvants.
• LEISHDNAVAX: Naked multiepitope DNA vaccine.
• ChAd63-KH: An adenovirus-based vaccine.
• *Leishmania major*/*Leishmania donovani* centrin–: A live, genetically attenuated vaccine.

This review of the literature is aimed at describing the clinical presentation, diagnosis, management, and outcomes of mucosal leishmaniasis in patients with primary oral cavity involvement, as well as those who develop oral manifestations later in the disease course.

## 2. Case Presentation

### 2.1. Case Report

In our study, we present the case of a 75-year-old, Caucasian immunocompetent man who was referred to our unit in February 2024, for developing oral manifestations of MCL diagnosed in 2017. In this case, HIV and other causes of immunodeficiency were excluded. Given the complexity of leishmaniasis and its global epidemiological significance, this study is aimed at characterizing the clinical presentation, diagnostic approach, and outcomes of MCL with oral manifestations.

In 2017, patient referred to the Dermatology Department of Parma Hospital for the presence of erythematous-purple plaques localized at the central portion of the face and neck. Initially treated with systemic corticosteroid therapy, disappearance of lesions but recurrence following discontinuation of systemic therapy. The diagnosis of CL was achieved in 2017 by histological examination of a biopsy from the neck skin and subcutaneous specimen. Histopathological description of the specimen showed chronic granulomatous lymphoplasmacytic and histiocytic dermatitis with the presence of *Leishmania* amastigotes. From the analysis of *Leishmania* DNA using polymerase chain reaction (PCR) technique, the result was positive.

At first, the patient was treated with Amphotericin B (three sessions, 3 mg/kg/day), but this did not lead to a substantial improvement in their condition. In May 2018, they started treatment with miltefosine (150 mg/day), but this was interrupted after 28 days due to difficulties in obtaining the medication, with partial benefit. Miltefosine treatment was resumed in December 2019 and again in November 2021, both times for 28 days. These treatments resulted in a noticeable improvement in the patient's clinical condition and absence of side effects.

In January 2020, the patient develops lesions in the hard palate, and biopsy was performed. Histopathologic analysis and PCR analysis resulted positive for *Leishmania*.

In November 2022, there was a recurrence of erythema and edema of the face, specifically at the nose, frontal region, supraciliary region, and lips. This was associated with dysphagia and burning in the oral cavity, which had been previously treated for MCL. Ultrasonographic examination revealed bilateral reactive lymphadenopathy but no major changes in the visceral organs. In January 2023, a further cycle of miltefosine at 150 mg/day for 28 days resulted in the resolution of clinical condition.

**Figure 1:**
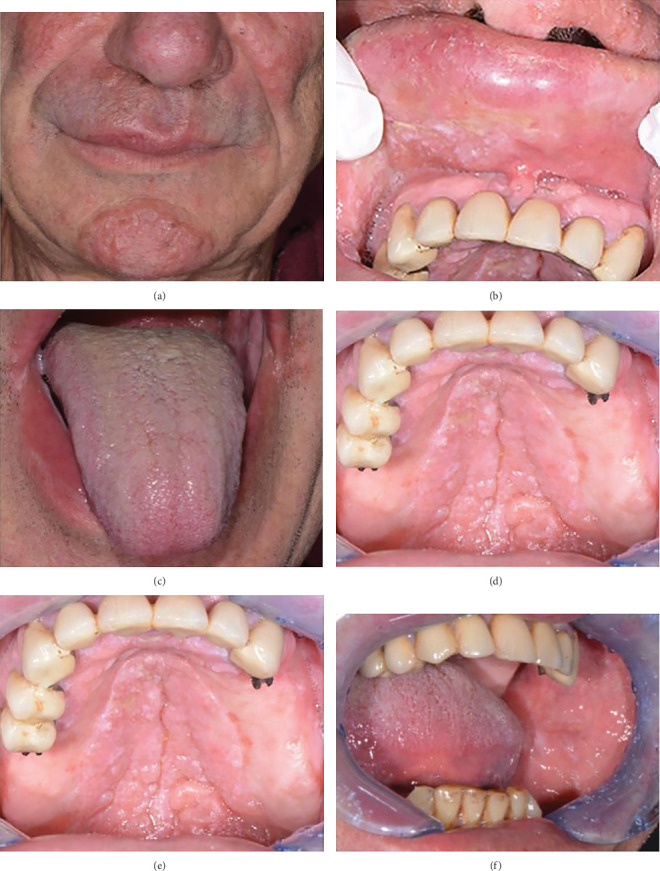
Clinical presentation of oral involvement. (a) Perioral erythema. (b) Nonpainful swelling of the upper lip. (c) Lingual erythema. (d–f) Leukokeratosis lesions of hardened consistency on the cheek mucosa and hard palate.

In February 2024, the patient is referred to our unit for the appearance of oral involvement. On clinical examination, the patient presented with leukokeratosis lesions of hardened consistency on the cheek mucosa, palate, and right side of the upper lip. Additionally, a nonpainful swelling of the upper lip was observed upon palpation (Figures 1a, 1b, 1c, 1d, 1e, and 1f).

Following the initiation of miltefosine 50 mg twice daily for 28 days in January 2024, a marked improvement in the clinical condition was observed in May 2024.

**Figure 2:**
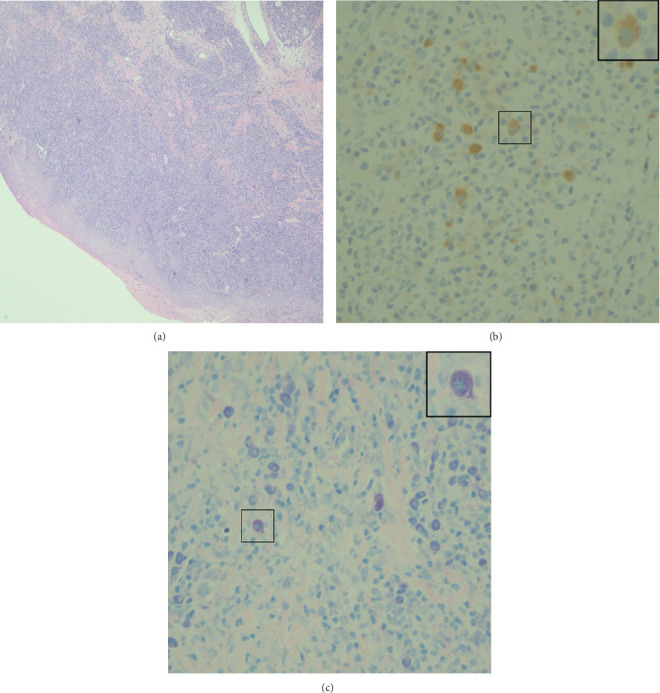
Case of mucocutaneous leishmaniasis. (a) Inflammation with lymphohistiocytic infiltrate H&E ×4. (b) CD1a immunohistochemical staining shows a CD1a-positive macrophage ×40. (c) Intracellular *Leishmania* amastigotes visualized with Giemsa stain ×40.

In February 2025, the patient underwent new incisional biopsy of the right genial mucosa; histopathological analysis confirmed the diagnosis of leishmaniasis (Figure 2). Hematoxylin and eosin (H&E) staining revealed an inflammatory infiltrate predominantly composed of lymphocytes and histiocytes (Figure 2a). Immunohistochemical staining for CD1a demonstrated the presence of a CD1a-positive macrophage within the lesion (Figure 2b). Furthermore, Giemsa staining clearly visualized numerous intracellular *Leishmania* amastigotes within macrophages (Figure 2c), providing direct evidence of parasitic infection.

### 2.2. Literature Review

Relevant studies reporting cases of patients with mucosal leishmaniasis with oral cavity involvement published between 1972 and February 2025 were identified through searches of the PubMed/Medline electronic database. A combined search strategy utilizing Medical Subject Headings (MeSH terms) and free-text keywords was employed: ((“leishmaniasis”[MeSH Terms] OR “leishmaniasis”[All Fields] OR “leishmaniases”[All Fields] OR “leishmaniasis vaccines”[MeSH Terms] OR (“leishmaniasis”[All Fields] AND “vaccines”[All Fields]) OR “leishmaniasis vaccines”[All Fields]) AND (“mouth”[MeSH Terms] OR “mouth”[All Fields] OR “mouths”[All Fields] OR “mouth s”[All Fields] OR “mouthed”[All Fields] OR “mouthful”[All Fields] OR “mouthfuls”[All Fields] OR “mouthing”[All Fields] OR (“mouth”[MeSH Terms] OR “mouth”[All Fields] OR “oral”[All Fields]) OR (“buccal”[All Fields] OR “buccally”[All Fields])).

Article inclusion in the review was determined through a two-stage evaluation process. First, titles and abstracts were independently screened by two reviewers (PDF and IG). Subsequently, potentially relevant articles underwent a full-text analysis. Discrepancies were resolved through discussion with a senior reviewer (PV).

Inclusion criteria for the title and abstract analysis were the following:

• Manuscripts published in English without any restriction of time.
• Articles with full text available.
• Patients with mucosal leishmaniasis with oral cavity involvement.
• Patients with MCL who subsequently developed oral manifestations.

Exclusion criteria for title and abstract analysis were as follows:

• Not original study (abstract, guidelines, and letters).
• Systematic reviews and review articles were not included.
• Manuscripts published not in English.
• Studies on leishmaniasis in nonhuman species.

For the reviewed articles, the following information was reviewed, selected, and extracted: age, sex, country of origin, immunodeficiency status, oral clinical presentation, treatment, follow-up, and outcomes.

### 2.3. Results of the Literature Review

**Figure 3:**
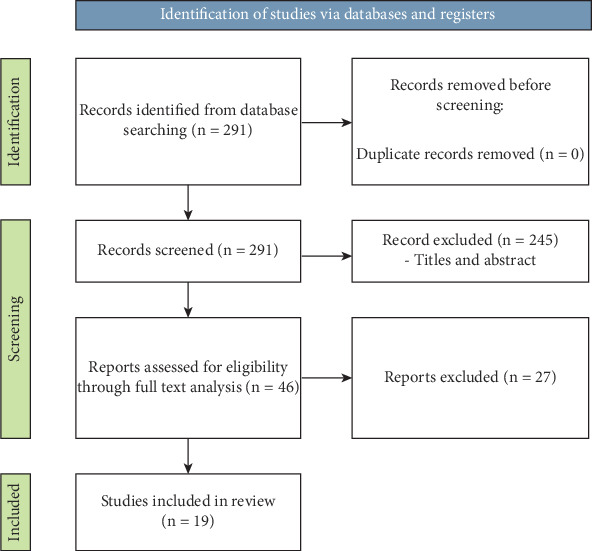
Research strategy.

A total of 291 citations of articles published, without restriction of time, were identified for inclusion in the review (Figure 3). Two hundred and forty-five articles were excluded following the screening of titles and abstracts. Full-text analysis was conducted on the remaining 46 articles, and 27 were further excluded as it was not possible to obtain the full article, were not written in English, or there was not oral involvement. Nineteen articles were included in the final review [15, 25–42].

The data collected from our research are summarized in Table 1.

Twenty-three patients (100% male) were evaluated; the age ranged from 28 to 94 years with a mean (±SD) age at the time of the onset of the disease of 55 (±19) years.

Palatal lesions were most common, affecting 10 patients (43%). The tongue and gingiva were the next most frequently affected sites, followed oral floor and buccal mucosa.

The oral lesions presented with nonspecific characteristics, including erythema, swelling, ulceration, or exophytic lesions.

The patients in this review received various treatments. Meglumine antimoniate was administered to 10 out of 23 patients (43.5%), Amphotericin B to 9 (39.1%), and miltefosine to 1 (4.3%). One out of 23 patients (4.3%) received a combination of meglumine antimoniate and miltefosine. The treatment drug could not be identified for two patients (8.7%).

Regarding treatment outcomes, 12 patients (52.2%) were completely cured, while the outcome was unevaluable in 5 (21.7%). Three patients (13%) showed clinical improvement, one (4.3%) developed VL, one (4.3%) relapsed after initial improvement, and one (4.3%) died due to disseminated disease.

## 3. Discussion

*Leishmania* parasites are divided into two dominant groups: Old World species are *L. major*, *Leishmania infantum*, and *Leishmania tropica*, and the New World species are *Leishmania amazonensis*, *Leishmania chagasi*, *Leishmania mexicana*, *Leishmania Viannia*, *Leishmania braziliensis*, and *Leishmania guyanensis*. Old World species predominantly cause self-limiting ulcers, whereas New World species can lead to severe, potentially fatal MCL [6].

With an estimated 600,000 to 1 million new cases annually, CL is the most common clinical manifestation of leishmaniasis. Eight countries—Afghanistan, Algeria, Brazil, Iran, Pakistan, Peru, Saudi Arabia, and Syria—account for 90% of global cases [5].

*Leishmania* species has two distinct phases in its life cycle [43]:

• Promastigote: Flagellated extracellular forms in sand fly's intestines.
• Amastigote: Unflagellated and obligate intracellular forms found in vertebrate hosts.

Virulent promastigotes, introduced into the mammalian skin by female sand flies, are rapidly internalized by mononuclear cells and transforming into the virulent amastigote form. Phagocytized amastigotes disseminate via the bloodstream and lymphatics, potentially leading to visceral or mucosal disease depending on the *Leishmania* species [44]. Whether the infection remains asymptomatic or progresses to symptomatic disease is influenced by a combination of host and parasite species-specific factors [3]. *Leishmania* has evolved several mechanisms to evade the host immune system. Several virulence factors, including LPG, GP63, and EF1-*α*, have been identified that interfere with the host immune response by inhibiting NF-*κ*B activation, modulating ROS and RNS production, and influencing the immune response toward a Th2 profile favorable to the parasite [45]. The relationship between the host and the leishmaniasis parasite is very complex and still not fully explained. Inflammation induced by the sand fly bite plays a crucial role, but the exact mechanisms by which the parasite manipulates the host's immune response to promote its own survival are still being studied [46]. Animals can host the leishmaniasis parasite, maintaining the life cycle of the disease. Dogs, rodents, marsupials, monkeys, and other mammals can be infected but do not always show symptoms. The dog is considered the main animal reservoir for *L. infantum*. Transmission of leishmaniasis can also occur through other routes, such as organ transplantation, blood transfusions, intravenous drug use, and congenital transmission, although these are less common [3].

Leishmaniasis presents with three main clinical manifestations, including cutaneous, mucosal, and visceral forms and some lesser prevalent clinical entities [7]. While some individuals remain asymptomatic, others may experience acute, subacute, or chronic disease. Leishmaniasis can have serious consequences in immunocompromised patients, particularly those with HIV [47]. Coinfection with *Leishmania* and HIV aggravates the infection, leading to higher parasite loads, more frequent relapses, and significant difficulties in treatment. Depending on the *Leishmania* species and the patient's immune status, HIV-positive individuals are more likely to develop MCL with initial oral lesions or VL with oral manifestations [32].

MCL, the least common form of the disease, is caused by *L. braziliensis* in the Americas and by *Leishmania aethiopica* in the Old World [48]. Mucosal involvement caused by New World *Leishmania* species occurs in 1%–10% of cases, typically developing 1–5 years after the healing of CL. However, in some instances, mucosal and skin lesions may appear simultaneously. Approximately 90% of these patients have a previous cutaneous scar. In rare cases, mucosal lesions can develop without any prior skin involvement because mucosal lesions typically develop months or years after [16].

ML often begins as erythema and ulceration, primarily in the nasal cavity, and progresses to septal perforation and destructive inflammatory lesions [34]. Lesions typically appear red or purple and can present as nodules, polypoid growths, or granular inflammation. Ulcerations may occur on the tongue, tonsils, lips, palate, and vocal cords [49]. Moreover, the clinical presentation can mimic other oral diseases, including leprosy, tuberculosis, hanseniasis, sarcoidosis, lymphoma, lupus vulgaris, squamous cell carcinoma (SCC), Langerhans cell histiocytosis, and other granulomatous infections [31]. While oral mucosal involvement is uncommon in patients with ML infection, affecting only 3%–5% of cases, it can still occur. Although any part of the oral mucosa can be involved, ulceration of the hard or soft palate is the most typical presentation. Oral leishmaniasis lesions typically manifest as granulomatous ulcers or vegetative growths, often accompanied by indurated swelling and hyperemia of the surrounding mucosa [27].

CL is the most common form of leishmaniasis, less severe and usually self-healing. Clinically, CL is classified into several distinct forms: localized, diffuse, disseminated (HIV positive or HIV negative), mucocutaneous, and post–kala-azar cutaneous. In CL, the most frequent symptom is the appearance of a nodule that, over time, develops into an ulcer. This process begins with a small papule that gradually grows and develops a central ulceration. The lesion has a characteristic shape and may be accompanied by other small surrounding lesions [50]. CL is caused by several species of *Leishmania*. In the Old World, the main species are *L. major* and *L. tropica*, while in Central and South America, there are *L. mexicana*, *L. amazonensis*, *L. guyanensis, Leishmania panamensis*, and *L. braziliensis* [7].

VL, also known as kala-azar, is the most severe form of the disease, characterized by infection of internal organs such as the liver, spleen, and bone marrow. *L. donovani* and *L. infantum* are the main etiological agents in the Old World, while *L. chagasi* is in the New World. Symptoms include fever, weight loss, anemia, hypergammaglobulinemia, and hepatosplenomegaly, often due to the high parasitic load. Hypersecretion of adrenocorticotropic hormone can cause skin ischemia [7]. Many cases are initially asymptomatic, but symptoms may occur later, especially in immunocompromised patients. In individuals infected with HIV, VL is recognized as an opportunistic infection [51]. The co-occurrence of opportunistic infections, including Kaposi sarcoma, cytomegalovirus, systemic mycoses, mycobacterial infections, and herpes simplex, is possible in immunocompromised individuals [32]. Severe immunosuppression is associated with atypical clinical manifestations, potential progression from cutaneous to visceral disease, and the possibility of concurrent cutaneous and visceral involvement [25].

The wide range of often nonspecific symptoms associated with leishmaniasis makes it difficult for physicians to perform an accurate diagnosis, and to date, there is no gold standard available for the diagnosis of active leishmaniasis [52].

Three primary methods are used for leishmaniasis diagnosis: parasitological examination (direct observation of parasites [like amastigotes] in stained smears or through parasite culture), molecular tests (primarily employing PCR to detect *Leishmania* DNA), and serological tests (indirect immunofluorescence assay [IFA], direct agglutination test [DAT], enzyme-linked immunosorbent assay [ELISA], and immunochromatographic tests [ICTs]) [53]. Serological tests for leishmaniasis, even using recombinant proteins, are not always accurate, and results vary greatly depending on the type of disease and the area. Serological tests using peptides seem to be more promising, because they may work better and give more reliable results, even if the parasite is different [53]. Accurate species-level identification of the *Leishmania* parasite, using methods like isoenzyme analysis or molecular techniques, is essential in cases of CL where mucosal leishmaniasis–causing species are circulating. This information directly impacts the clinical management of the patient [44]. Diagnosis of VL relies on serological tests to detect antibodies against the parasite. Diagnosis of CL is usually achieved by microscopic examination of tissue samples or smears for the presence of the parasite.

All individuals diagnosed with mucosal leishmaniasis should undergo systemic antileishmanial treatment to prevent morbidity and mortality, and a complete examination of the naso-oropharyngeal/laryngeal mucosa should be conducted [52]. Unlike CL, mucosal leishmaniasis does not heal spontaneously and can lead to progressive tissue destruction, disfigurement, and even death, potentially caused by aspiration pneumonia or respiratory obstruction [3]. Leishmaniasis treatment can be quite challenging, and it is influenced by numerous factors, including the clinical presentation, geographical region, and the specific parasite species [54]. It faces significant challenges due to the parasite's ability to evade the host immune response. Currently, no effective vaccine exists, and traditional treatments present limitations, including toxicity, high cost, prolonged administration periods, adverse side effects, and the emergence of drug resistance [55]. A 2019 Cochrane review indicates that antimonial drugs, particularly meglumine antimoniate, and oral miltefosine demonstrate the most significant improvement in clinical outcomes for American CL and MCL; however, evidence certainty was generally moderate or low due to methodological limitations, hindering definitive conclusions [18].

According the Clinical Practice Guidelines by the Infectious Diseases Society of America (IDSA) and the American Society of Tropical Medicine and Hygiene (ASTMH), the traditional options for ML are as follows: pentavalent antimonial (Sb^V^) compound (20 mg Sb^V^/kg daily, IV or IM, for 28–30 days) or with Amphotericin B deoxycholate (0.5–1.0 mg/kg per dose, IV, daily or every other day, for a cumulative total of ~20–45 mg/kg). Lipid formulations of Amphotericin B (typically, liposomal Amphotericin B [L-AmB], with a cumulative total dose ranging widely from ~20 to 60 mg/kg), as well as the oral agent miltefosine (~2.5 mg/kg per day [maximum, 150 mg/day] for 28 days) [52].

The clinical efficacy of these treatments can vary considerably based on the infection, clinical manifestation, and the specific *Leishmania* species involved [44]. Recently, immunotherapy, either alone or in conjunction with chemotherapy, has been investigated as a potential alternative to conventional treatments. By stimulating a targeted and specific immune response against the parasite, immunotherapy has shown promise in effectively treating patients who do not respond to conventional drugs [56].

The treatment of the patient described in this case report with miltefosine is consistent with existing literature supporting the efficacy and safety of this agent in managing MCL [18]. Clinical studies have documented cure rates ranging from 76% in infections caused by *L. braziliensis* to 83% when species identification is not available. Notably, in the Old World, individual case studies have reported cure rates as high as 100% [57]. These findings support the therapeutic choice in our case and highlight miltefosine as a reliable option in similar clinical scenarios. However, its limited availability and high cost despite being off-patent and lacking generic formulations continue to restrict its use in the endemic areas [58].

In this study, we presented a case of leishmaniasis with oral cavity involvement, an uncommon but potentially serious clinical manifestation of this disease. The simultaneous presence of perioral and intraoral lesions in an immunocompetent patient is an unusual clinical finding. Such presentations are more commonly associated with immunosuppression, especially HIV infection [25, 59, 60]. In this case, HIV and other causes of immunodeficiency were excluded. This case highlights the importance of considering oral leishmaniasis in the differential diagnosis of persistent ulcerative lesions of the oral cavity even in immunocompetent individuals. The management of oral leishmaniasis requires a multidisciplinary approach and customized therapy, adapted to the clinical and immunological characteristics of each patient. In conclusion, the presented case highlights the complexity of diagnosis and treatment of leishmaniasis disease and the need for accurate and early diagnosis to ensure an optimal patient management.

## Figures and Tables

**Table 1:** Epidemiology, clinical features, immunodeficiency status, treatment, outcome and follow-up. NI, no information available.

**Authors**	**Gender, age**	**Country**	**Oral lesions**	**Immune competent**	**Other mucosal/cutaneous site involvement**	**Treatment**	**Outcome and follow-up**
Cascio et al. [25]	M, 35	Iran	Multiple erosive lesions on the gingival mucosal; papular lesion on the left maxillary area; erythematous and painful tongue	No: HIV+	Yes	Liposomal Amphotericin B	Alive after 24 months; no relapse
Bajgai et al. [26]	M, 76	Bhutan	Large ill-defined vegetative mass on the anterior palate and the left buccal mucosa with maxillary gingival hyperplasia	Yes	No	Liposomal Amphotericin B	After the 9th infusion of Amphotericin B, the patient refused any further injections. At this point, there was a 50% posttreatment resolution of the lesions
Botelho et al. [27]	M, 57	Brazil	Indurated and ulcerated nodule on tongue dorsum	Yes	No	Meglumine antimoniate	13 years after treatment, a painless nodular fibrous tissue scar persisted on the tongue dorsum
De Ruiter et al. [28]	M, 40	Morocco	Maxillary severe periodontitis and atypical mucosal lesions on the hard palate	Prednisone and methotrexate for rheumatoid arthritis	No	Liposomal Amphotericin B	Three months, healing
Van Damme et al. [29]	M, 85	Netherlands	Hard palate ulcer	Yes	No	NI	NI
Almeida et al. [30]	M, 41	Brazil	Ulcerative lesions on the upper alveolar ridge mucosa/gingiva and the hard palate	Yes	No	Meglumine antimoniate	4 years, no relapse
Dos Santos et al. [31]	M, 80	Brazil	• Granulomatous ulceration on the upper lip, soft and hard palate	No	No	Meglumine antimoniate	Complete remission, 12 years
	M, 62	Brazil	• Ulcerated irregular lesions of upper alveolar ridge and hard palate mucosa	No	Yes	Meglumine antimoniate	Complete remission
Celentano et al. [15]	M, 50	Italy	Widespread exophytic mass of the left buccal mucosa	Yes	No	Amphotericin B	After 5 months, the patient had a visceral recurrence
Rosa et al. [32]	M, 35	Brazil	Erythematous, bleeding, and ulcerative lesions extended from the anterior inferior gingival mucosa to the lower anterior and posterior alveolar ridges	HIV+	No	Amphotericin B	NI
Pellicioli et al. [33]	M, 71	Brazil	Multiple ulcerated nodules on hard and soft palate ulcer	NI	No	Liposomal amphotericin	3 weeks, complete healing
García de Marcos et al. [34]	M, 70	Spain	• Hardened and painful ulceration with irregular margins on the floor of the mouth	HIV−	NI	Meglumine Antimoniate	• Healing, 10 years
	M, 41		• Painful ulceration on cheek mucosa	HIV+	NI	Meglumine antimoniate	• Healing, 1 year
	M, 50		• Bleeding and painful granulomatous, excrescent lesion on the upper alveolar ridge and palatal	HIV+	NI	NI	• NI
Chong et al. [35]	M, 62	NI	Swollen tongue with yellow-white ulcerated lesions	Yes	No	Liposomal Amphotericin B	Healing, 1 year
Mehlig et al. [36]	M, 71	Italy	Right-sided tongue swelling	Yes	No	Meglumine antimoniate	Relapse after 3 years
Palmeiro et al. [37]	M, 75	Brazil	Granular area affecting the hard and soft palate, uvula, and lower gingiva	Yes	No	Meglumine antimoniate	30 months, complete healing
Shirian et al. [38]	M, 34	Iran	Diffuse yellowish white erosions on buccal floor	Yes	Yes	Amphotericin B	1 week, complete healing
Cruz et al. [39]	M, 60	Brazil	Papillary lesions on the soft palate and uvula	Yes	Yes	Meglumine antimoniate	3 years, complete healing
	M, 94	Brazil		Yes	Yes	Liposomal Amphotericin B	Died because of generalized infection
Milián et al. [40]	28	NI	Exophytic lesions on hard palate	No: HIV+	No	Meglumine antimoniate	NI
Salam et al. [41]	M, 40	Bangladesh	Granulomatous nodules of tongue and perioral mucosal	NI	Yes	Miltefosine	Clinical improvement, 3 months
Mortazavi et al. [42]	M, 28	Iran	Erythematous–violaceous plaque on hard palate	Yes	Yes	Combination of miltefosine and meglumine antimoniate	Clinical improvement, 1 month

## Data Availability

Data are available on request from the authors.
